# Multiple Roles of the Low-Affinity Calcium Uptake System in *Drechslerella dactyloides*, a Nematode-Trapping Fungus That Forms Constricting Rings

**DOI:** 10.3390/jof9100975

**Published:** 2023-09-28

**Authors:** Xiaozhou Zhao, Yani Fan, Liao Zhang, Weiwei Zhang, Meichun Xiang, Seogchan Kang, Shunxian Wang, Xingzhong Liu

**Affiliations:** 1 State Key Laboratory of Medicinal Chemical Biology, Key Laboratory of Molecular Microbiology and Technology of the Ministry of Education, Department of Microbiology, College of Life Science, Nankai University, Tianjin 300071, China; 2 State Key Laboratory of Mycology, Institute of Microbiology, Chinese Academy of Sciences, Beijing 100101, China; 3 University of Chinese Academy of Sciences, Beijing 100049, China; 4 Department of Plant Pathology & Environmental Microbiology, The Pennsylvania State University, University Park, PA 16802, USA; sxk55@psu.edu

**Keywords:** calcium signaling, constricting ring, *Drechslerella dactyloides*, fungal carnivorism, RNAi, trap formation

## Abstract

(1) Background: the low-affinity calcium uptake system (LACS) has been shown to play a crucial role in the conidiation and formation of adhesive nets and knobs by nematode-trapping fungi (NTF), but its involvement in the formation of constricting rings (CRs), mechanical traps to capture free-living nematodes, remains unexplored. (2) Methods: we investigated the function of two LACS genes (*DdaFIG_1* and *DdaFIG_2*) in *Drechslerella dactyloides*, an NTF that forms CRs. We generated single (*DdaFIG_1Ri* and *DdaFIG_2Ri*) and double (*DdaFIG_1,2Ri*) knockdown mutants via the use of RNA interference (RNAi). (3) Results: suppression of these genes significantly affected conidiation, trap formation, vegetative growth, and response to diverse abiotic stresses. The number of CRs formed by *DdaFIG_1Ri*, *DdaFIG_2Ri*, and *DdaFIG_1,2Ri* decreased to 58.5%, 59.1%, and 38.9% of the wild-type (WT) level, respectively. The ring cell inflation rate also decreased to 73.6%, 60.6%, and 48.8% of the WT level, respectively. (4) Conclusions: the LACS plays multiple critical roles in diverse NTF.

## 1. Introduction

**Figure 1:**
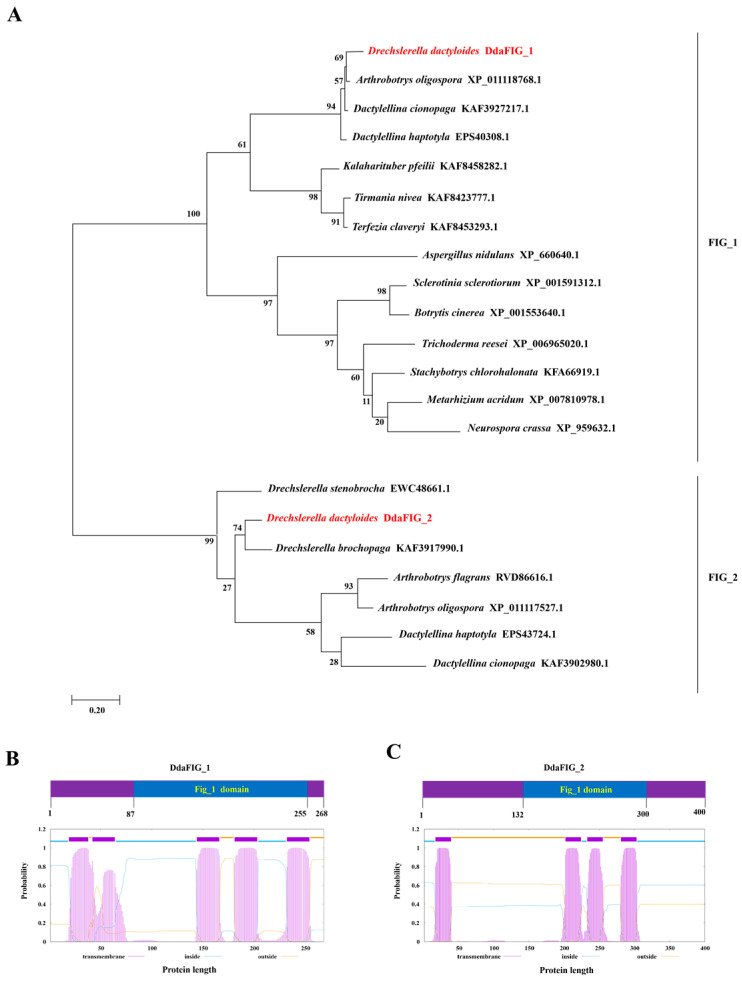
Phylogenetic analysis and transmembrane structure prediction of the DdaFIG_1 and DdaFIG_2 proteins. (**A**) Phylogenetic analysis of DdaFIG_1, DdaFIG_2, and their fungal homologs. The origin and GenBank accession of the proteins included are noted. The predicted transmembrane regions of (**B**) DdaFIG_1 and (**C**) DdaFIG_2 are shown. The conserved FIG_1 domain is marked.

All organisms employ a network of signaling pathways to modulate their growth and development in response to external stimuli and internal signals. Calcium ion (Ca^2+^) functions as a ubiquitous intracellular signaling molecule in eukaryotes and controls diverse cellular and developmental processes by modulating the cellular localization or activity of diverse Ca^2+^-binding proteins (CBPs) and CBP-interacting proteins in response to external stimuli. This modulation is achieved by the adjusting spatial and temporal distribution patterns of cytoplasmic Ca^2+^ via influxes and effluxes of Ca^2+^ to and from the extracellular environment and internal Ca^2+^ stores through the action of ion channels, pumps, and transporters on plasma and organellar membranes. In filamentous fungi, Ca^2+^-mediated signaling has been shown to regulate multiple processes, including spore production and germination and hyphal tip growth and branching [1,2,3]. Fungi utilize two distinct Ca^2+^ uptake systems to acquire extracellular Ca^2+^, with the high-affinity calcium uptake system (HACS) being active under low external Ca^2+^ concentrations and the low-affinity calcium uptake system (LACS) becoming active under high external Ca^2+^ concentrations [4]. Only one gene named *FIG_1* (Factor-Induced Gene 1) encodes the LACS in most fungi [5].

The FIG_1 protein contains four putative membrane-spanning domains and a conserved claudin motif (GΦΦGXC(n)C, where “Φ” corresponds to a hydrophobic amino acid and “n” means any number of amino acids) [6]. The *FIG_1* protein was first found in *Saccharomyces cerevisiae* and is involved in cell fusion during the mating process, and its deletion results in incomplete fusion, a defect that can be restored by adding exogenous Ca^2+^ [7]. In filamentous fungi, multiple studies have shown the involvement of *FIG_1* in regulating various processes. Deletion of *FIG_1* in *Fusarium graminis* resulted in slower growth, reduced sporulation, diminished virulence, and inhibited perithecia development [8]. The loss of *FIG_1* in *Neurospora crassa* affected sexual development [9], and its knockout in *Aspergillus nidulans* decreased vegetative growth, reduced conidia production, and inhibited self-mating [10].

Plant parasitic nematodes invade plants and cause severe crop yield losses worldwide. It is well recognized that traditional nematicides suffer from their side-effects, including environmental and human toxicity, calling for effective alternatives. Nematode-trapping fungi (NTF) have evolved multiple trapping devices, including two-dimensional (2-D) adhesive traps like knobs, columns, and non-constricting rings, and three-dimensional (3-D) adhesive traps like networks, and mechanical traps like constricting rings (CRs), to capture nematodes, which makes them promising biological control agents against plant parasitic nematodes [11]. Understanding the predation mechanism of NTF is important for exploiting their predation strategies for controlling nematodes. Most NTF adopt two lifestyles, saprophytism and predatism. Most NTF differentiate traps in response to signals from their surrounding environment, including living nematodes, nematode extract, nemin, amino acids, small peptides, bacteria, abscisic acid, and ascarosides [12]. Comparative genomic analyses [13,14], transcriptomics [15], proteomics [16,17,18], and metabolomics [19] have helped better understand the molecular mechanism of predation by NTF. However, a comprehensive understanding of how traps form and operate still requires more studies.

Comparative genomic analyses of NTF have unveiled the presence of two genes homologous to *FIG_1*, both of which appear to participate in the formation of predatory traps [20,21]. The 3-D trap-forming fungus *Arthrobotrys oligospora* encodes two proteins homologous to *FIG_1*, named *AoFIG_1* and *AoFIG_2*. Deletion of the gene encoding *AoFIG_1*, the ortholog of known fungal *FIG_1* protein, reduced the trap formation by 90%, while deletion of the gene for *AoFIG_2* led to a 44% decrease in vegetative growth and completely blocked trap formation and conidiation [20]. One ortholog of the gene for *AoFIG_2* is also present in other NTF, including the 2-D adhesive knob-forming *Dactylellina haptotyla* [21]. Suppression of *DhFIG_2* expression in *D. haptotyla* via RNA interference (RNAi) resulted in a 90% reduction in conidia formation and a 34% reduction in the number of knobs [21].

Our study aimed to investigate whether *DdaFIG_1* and *DdaFIG_2*, the *AoFIG_1* homologs in CR-forming *D. dactyloides*, also play critical roles in CR formation and its function. *Drechslerella dactyloides* forms CRs, consisting of two stalk cells and three ring cells, to capture nematodes by inflating ring cells. Although the initial establishment of transformation protocols was challenging [22,23,24,25], we successfully investigated the function of *DdaFIG_1* and *DdaFIG_2* in *D. dactyloides* using RNAi. We characterized their role in vegetative growth, conidiation, trap-formation, and response to diverse abiotic stresses through the use of RNAi and *Agrobacterium tumefaciens*-mediated transformation (ATMT).

## 2. Materials and Methods

### 2.1. Strains and Growth Conditions

The *D. dactyloides* isolate CGMCC3.20198 was cultured from a soil sample from Motuo, Tibet, China, by us. This strain and its mutants were cultured on potato dextrose agar (PDA, BDTM, USA), tryptone glucose (TG, BDTM, USA) agar, and corn meal agar (CMA, BDTM, USA) at 28 °C. For conidiation, they were cultured on CMA plates supplemented with 2 g/L KH_2_PO_4_. Water agar plates were used to induce trap formation by introducing *Caenorhabditis elegans*. For stress assays, they were cultured on PDA with different concentrations of chemical stressors at 28 °C. *C. elegans* was maintained on nematode growth medium (NGM) agar plates at 23 °C and fed with *Escherichia coli* OP50. *E. coli* OP50 was cultured in Luria-Bertani (LB) medium at 37 °C. *E. coli* DH5α was used for plasmid construction and maintenance, and was cultured in LB at 37 °C. *A. tumefaciens* strain AGL-1 was used for transforming *D. dactyloides* and was cultured in LB at 28 °C. Transformants of *E. coli* DH5α and *A. tumefaciens* AGL-1 were selected using agar plates supplemented with 100 μg/mL kanamycin sulfate (Sangon Biotech, Shanghai, China). The antibiotic solution was filtered with 0.22 μm membrane before use.

### 2.2. Sequence and Phylogenetic Analyses of DdaFIG_1 and DdaFIG_2

Data from the whole genome shotgun (WGS) project of *D. dactyloides* CGMCC3.20198 were deposited at GenBank with the accession number JAGTWJ000000000 (BioSample SAMN18837316) and are accessible under the BioProject number PRJNA723920. Two *FIG_1* homologs encoded by *D. dactyloides* were identified via BLASTP using the amino acid sequences of FIG_1 encoded by *Aspergillus fumigatus*, *Botrytis cinerea*, and *Schizosaccharomyces pombe* as queries. The isoelectric points and molecular weights of the DdaFIG_1 and DdaFIG_2 proteins were predicted using the online software ExPASy-ProtParam tool (https://web.expasy.org/protparam, accessed on 10 August 2023), and conserved functional domains were identified using SMART (http://smart.embl-heidelberg.de, accessed on 10 August 2023) and InterProScan (http://www.ebi.ac.uk/interpro, accessed on 10 August 2023). Additional FIG_1 orthologs encoded by other fungi were retrieved from the NCBI database using BLASTP. A phylogenetic tree based on FIG_1 amino acid sequences was constructed using the MEGA7 software package.

### 2.3. RNAi Vector Construction and Transformation

The construction of RNAi vectors for suppressing the expression of *DdaFIG_1*, *DdaFIG_2*, or both was performed as previously reported [21]. The DNAMAN software was used to align the gene sequences to identify targets. The hairpin structure was designed, and individual DNA fragments were synthesized. After digesting the synthesized constructs with *Hpa*I and *Nhe*I, they were ligated to the plasmid pAg1-H3-R1 digested with *Hpa*I and *Nhe*I to build DdaFIG_1RNAi and DdaFIG_2RNAi, respectively (Figure S1). The vector pAg1-H3-R2 designed to suppress both genes was constructed by inserting a fragment containing TrpC-IT-TrpC, which was amplified from plasmid pAg1-H3-R1 (Figure S1). Then, the *DdaFIG_2* target sequence (released using *Age*I and *Mfe*I) was cloned into plasmid pAg1-H3-R2. The *DdaFIG_1* target sequence (released using *Hpa*I and *Nhe*I) was then cloned into this plasmid to form DdaFIG_1,2RNAi. The resulting plasmids were transformed into *A. tumefaciens* AGL-1 (Biomed, Beijing, China).

The ATMT method was used to introduce these RNAi constructs into the genome of *D. dactyloide*s [22]. Conidia (ca. 10^6^) of *D. dactyloides* harvested from 14-day-old cultures on CMA were co-cultured with *A. tumefaciens* AGL-1 harboring each RNAi binary vector for 7 days in IM medium (pH was adjusted to 5.2~5.6 with MES monohydrate (Sangon Biotech, Shanghai, China)). The co-cultures were then overlaid with PDA medium containing 100 μg/mL hygromycin B (Leagene, Beijing, China) and 400 μg/mL cefotaxime sodium (Henghuibio, Beijing, China) for selection. Potential transformants growing on the selection medium were transferred to new selection medium. To confirm the stability of the transformants, cultures derived from single conidia were transferred for 5 generations on PDA plates supplemented with 100 μg/mL hygromycin B. Finally, the stable transformants were further analyzed by molecular methods.

### 2.4. Extraction of Genomic DNAs and Total RNAs

Positive transformants were confirmed by checking the presence of the hygromycin B resistance gene and the decreased level of the *DdaFIG_1* and *DdaFIG_2* transcripts. To identify transformants containing the hygromycin resistance gene, their genomic DNA was extracted using Fungi Genome DNA Extration Kit (Solarbio, Beijing, China) [26]. Mycelia (about 50~100 mg) were scraped from PDA plates of the WT and transformants carrying each RNAi construct using a small sterilized spoon and transferred into 1.5 mL tubes. Mycelia were pulverized using a tissue grinder containing 2.5 mm small steel balls at 70 Hz/min for 1 min for three times. The presence of the hygromycin B resistance gene was confirmed by PCR using primers HYG-540F/HYG-540R (Table S1); 2 × Rapid Taq Master Mix (Vazyme, Nanjing, China) was used for the PCR reaction.

Total RNAs were extracted from the WT and its transformants using TRIzol (Invitrogen TM, Carlsbad, CA, USA) as previously described to measure the levels of *DdaFIG_1* and *DdaFIG_2* transcripts [27]. Conidia of the WT and transformants were placed on WA plates, covered with cellophane membrane, and incubated for 5 days. Their mycelia were scraped using a sterilized spatula and collected in RNase-free 1.5 mL tubes for RNA extraction. Liquid nitrogen, chloroform, isopropyl alcohol, RNase-free water, and RNase-free 75% ethanol were used.

### 2.5. Comparison of Mycelial Growth and Conidial Production

The growth rates of the WT and its transformants under different nutritional conditions were compared by inoculating discs (5 mm in diameter) punched from the edges of 14-day-old colony on CMA plate onto PDA, TG agar, and CMA plates. During 12 days of incubation at 28 °C, colony diameters were measured every two days. Conidia from the 14-day-old cultures on CMA plates were harvested using distilled water containing 0.1% Tween-20. The number of conidia produced was counted using a hemocytometer.

### 2.6. Analyses of Mycelial Growth under Different Stresses

Discs (5 mm in diameter) punched from the edges of 14-day-old cultures on CMA plates were placed on the center of PDA plates supplemented with various stress-inducing agents, including (a) Congo red at 0.1 mg/mL, 0.2 mg/mL, and 0.4 mg/mL; (b) SDS at 0.01% and 0.02%; (c) NaCl and KCl at 0.1 M, 0.2 M, and 0.3 M; (d) sorbitol at 0.2 M, 0.5 M, and 0.8 M; and (e) H_2_O_2_ at 0.001% and 0.003%. Colony diameters were measured after 14 days of incubation at 28 °C. Evaluation of the effect of low temperature was assessed by incubating cultures at 15 °C. The relative growth rate caused by each stress was calculated using the equation Dt/Dc × 100%, where Dc and Dt refer to the diameters of the unstressed (control) and stressed colonies, respectively.

### 2.7. Measurement of Constricting Ring Formation and Ring Cell Inflation after Introducing Nematodes

To compare the ability of the WT and its transformants to form CRs, ca. 1000 conidia from each strain were inoculated onto 2% WA plates and incubated at 28 °C. After 3 days incubation, approximately 1000 *C. elegans* larvae per plate were inoculated. The numbers of CRs and inflated CRs within a 4 cm^2^ area of each colony were counted using a microscope at 16 h and 24 h after nematode inoculation.

### 2.8. Real-Time PCR (RT-PCR) Analysis

Total RNAs extracted from the WT and its transformants were reversely transcribed into cDNA using a FastKing RT Kit with gDNase (Vazyme, Nanjing, China). The PCR reactions were performed using SYBR Green RT-PCR Master Mix (Vazyme, Nanjing, China), and the β-tubulin gene was used as an internal standard. Sequences of the primer pairs used (DdaFIG_1-RT-F/R, DdaFIG_2-RT-F/R and Tublin-RT-F/R) are shown in Table S1. To calculate the relative transcriptional level, the 2^−ΔΔCt^ method was used [28]. All analyses were performed in triplicate.

### 2.9. Statistical Analysis

Experimental data were statistically analyzed using GraphPad Prism version 5.00 (GraphPad Software, San Diego, CA, USA) and presented as mean ± SD. The *p*-values < 0.05 using Student’s *t*-test were considered statistically significant.

## 3. Results

### 3.1. Identification of Two FIG1 Homologs Encoded by D. dactyloides

The FIG_1 protein of *S. cerevisiae* contains four transmembrane regions and is involved in Ca^2+^ transport [7]. In the genome of *D. dactyloides*, we discovered two genes homologous to *FIG_1*, named as *DdaFIG_1* and *DdaFIG_2*. The *DdaFIG_1* gene is 1405 bp-long and encodes a 29.3 kDa protein with 268 amino acids and an isoelectric point (pI) of 4.95. The *DdaFIG_2* gene is 1257 bp-long, encoding a 42 kDa protein with 400 amino acids and a pI of 9.26. Phylogenetic analysis of DdaFIG_1, DdaFIG_2, and their fungal homologs, including those encoded by plant pathogenic fungi (*Sclerotinia sclerotiorum*, *Botrytis cinerea*) and mycoparasitic fungus *Trichoderma reesei*, showed that the protein with the highest identity to DdaFIG_1 is that encoded by *A. oligospora*, another NTF (Figure 1A). The protein with the highest identity to DdaFIG_2 is the one encoded by *D. stenobrocha*, and its ortholog was found only in NTF (Figure 1A). TMHMM-Scan predicted that DdaFIG_1 and DdaFIG_2 are transmembrane proteins containing the conserved FIG_1 domain, with DdaFIG_1 and DdaFIG_2 having five and four transmembrane regions, respectively (Figure 1B,C).

### 3.2. Suppression of the Expression of DdaFIG_1 and DdaFIG_2, Individually and Simultaneously, via RNAi

**Figure 2:**
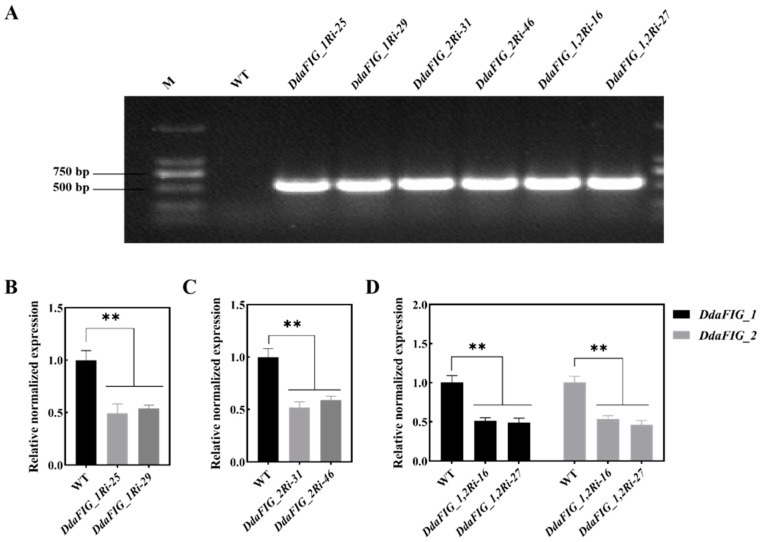
Analyses of the transformants containing an RNAi construct for suppressing the expression of *DdaFIG_1*, *DdaFIG_2*, or both. (**A**) Verification of the presence of the hygromycin B resistance gene; (**B**) expression levels of *DdaFIG_1* in two independently isolated transformants containing the DdaFIG_1 RNAi construct; (**C**) expression levels of *DdaFIG_2* in transformants containing the DdaFIG_2 RNAi construct; (**D**) expression levels of *DdaFIG_1* and *DdaFIG_2* in transformants containing the DdaFIG_1,2 RNAi construct. ** *p* < 0.01.

We utilized a combination of RNAi and ATMT to suppress the expression of *DdaFIG_1* and *DdaFIG_2*. The presence of the hygromycin B resistance gene in putative transformants was verified (Figure 2A, Table S1). The transcript levels in the single gene suppression (*DdaFIG_1Ri-25*, *DdaFIG_1Ri-29*, *DdaFIG_2Ri-31*, *DdaFIG_2Ri-46*) and double gene suppression (*DdaFIG_1,2Ri-16*, *DdaFIG_1,2Ri-27*) transformants were significantly reduced compared to the wild-type (WT) strain (Figure 2B–D; Tables S2–S4), confirming the successful RNAi of *DdaFIG_1* and *DdaFIG_2* in *D. dactyloides*.

### 3.3. Effects of Knocking Down DdaFIG_1 and DdaFIG_2 on Vegetative Growth and Sporulation

**Figure 3:**
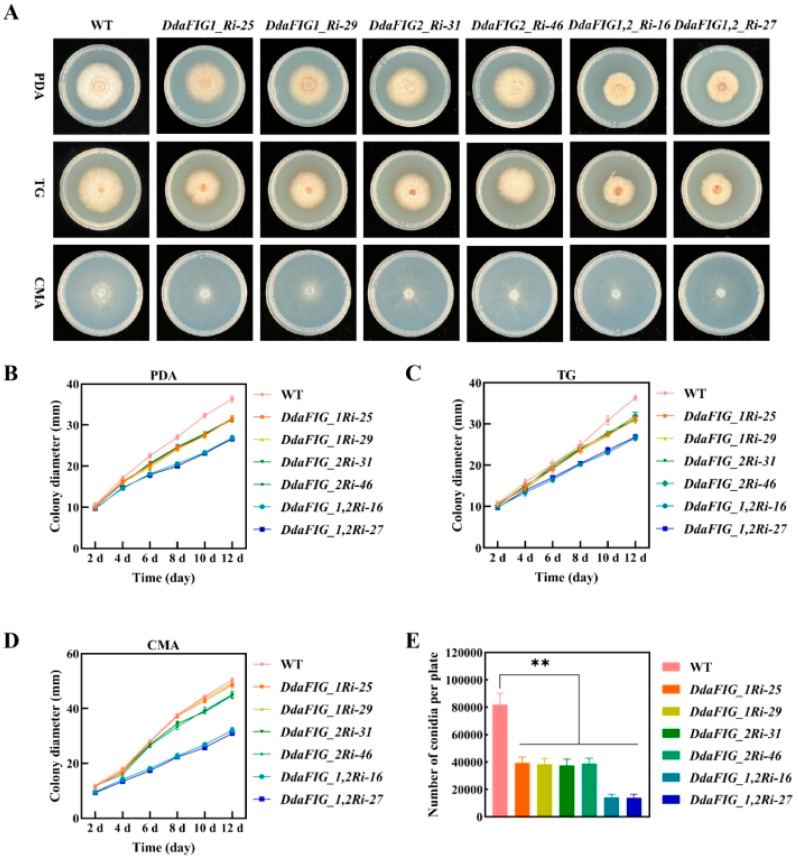
Colony morphology, growth rates, and conidiation of the WT and selected DdaFIG_1Ri, DdaFIG_2Ri, and DdaFIG_1,2Ri transformants. (**A**) Colony morphology on PDA, TG, and CMA. Colony growth rates on (**B**) PDA, (**C**) TG medium, and (**D**) CMA. (**E**) Conidial production on CMA. ** *p* < 0.01.

Growth characteristics on PDA, TG agar, and CMA were used to investigate the effect of knocking down *DdaFIG_1* and *DdaFIG_2* on vegetative growth and sporulation. The average growth rates of transformants containing DdaFIG_1Ri on PDA and TG agar were 87% and 85.5% of the WT, respectively, but were comparable on CMA (Figure 3A–C). The sporulation capacity of DdaFIG_1Ri transformants was only 47.6% of the WT level on CMA (Figure 3D, Table S5). Similarly, transformants containing DdaFIG_2Ri exhibited reduced growth rates (87%, 86.7%, and 89.1% of the WT on PDA, TG, and CMA, respectively) and sporulation (46.6% of the WT on CMA) (Figure 3A–D, Table S5). Transformants containing DdaFIG_1,2Ri displayed a more pronounced reduction in growth rates (74%, 73.5%, and 62.6% on PDA, TG, and CMA, respectively) and sporulation (17.5% of the WT on CMA) (Figure 3A–D, Table S5).

### 3.4. Effect of Knocking Down DdaFIG_1 and DdaFIG_2 on CR Formation and Ring Cell Inflation

**Figure 4:**
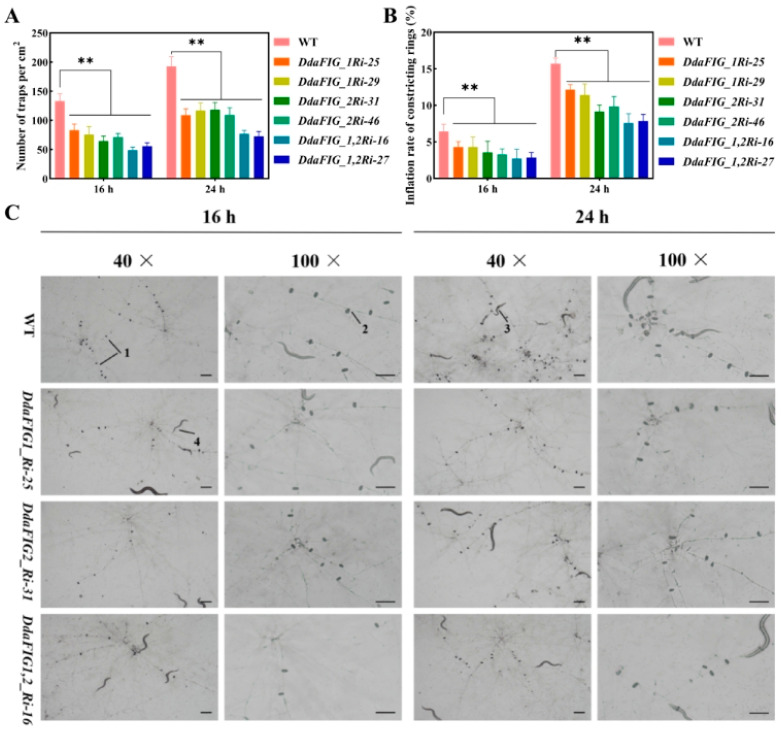
Trap formation and inflation of the WT strain and selected DdaFIG_1Ri, DdaFIG_2Ri, and DdaFIG_1,2Ri transformants. (**A**) Numbers of CRs formed and (**B**) CR inflation rates at 16 h and 24 h after introducing nematodes to culture plates. (**C**) Traps imaged under 40× and 100× magnification. Bar = 100 μm. ** *p* < 0.01.

Knocking down *DdaFIG_1* and *DdaFIG_2*, individually and simultaneously, caused a significant reduction in trap formation and ring cell inflation. The WT strain produced about 133/cm^2^ and 193/cm^2^ CRs 16 h and 24 h after introducing nematodes, respectively (Figure 4A, Table S6). Transformants containing DdaFIG_1Ri produced 80/cm^2^ and 113/cm^2^ CRs 16 h and 24 h after introducing nematodes (60.2% and 58.5% of the WT level), respectively (Figure 4A, Table S6). Similarly, transformants containing DdaFig_2Ri produced 68/cm^2^ and 114/cm^2^ CRs 16 h and 24 h after introducing nematodes (51.1% and 59.1% of the WT level), respectively (Figure 4A, Table S6). Those containing DdaFIG_1,2Ri exhibited more severe reductions, producing about 53/cm^2^ and 75/cm^2^ CRs 16 h and 24 h after introducing nematodes (39.8% and 38.9% of the WT level), respectively (Figure 4B, Table S7).

Ring cell inflation of these transformants also exhibited similar trends. The percentages of inflated traps of DdaFIG_1Ri, DdaFIG_2Ri, and DdaFIG_1,2Ri transformants were 71.6%, 53.8%, and 43% of the WT level, respectively, 16 h after induction (Figure 4B, Table S7) and 73.6%, 60.6%, and 48.8% of the WT level, respectively, 24 h after induction (Figure 4B, Table S7).

### 3.5. Effect of Knocking Down DdaFIG_1 and DdaFIG_2 on Abiotic Stress Response

**Figure 5:**
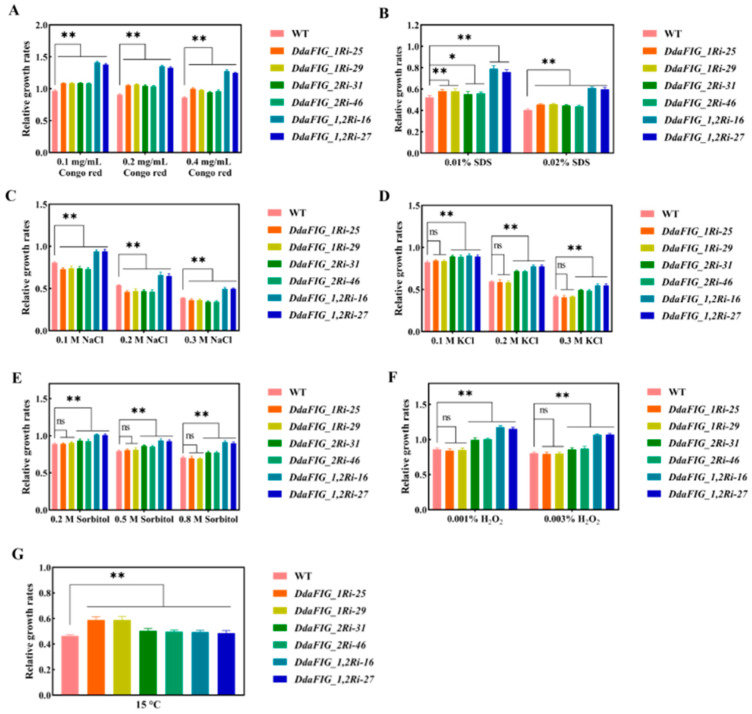
Growth rates of selected DdaFIG_1Ri, DdaFIG_2Ri and DdaFIG_1,2Ri transformants relative to the WT strain under abiotic stresses. Relative growth rates on PDA containing (**A**) Congo red, (**B**) SDS, (**C**) NaCl, (**D**) KCl, (**E**) sorbitol, (**F**) H_2_O_2_, and at (**G**) 15 °C. * means *p*-values < 0.05, ** means *p*-values < 0.01, ns means no significance, two-tailed *t*-test, *n* = 7.

Congo red, a cell wall inhibitor that specifically binds to *β*-1,3-glucose, and SDS, a surfactant that removes lipids associated with the cell wall, were chosen as cell wall stress reagents. Compared with the WT strain, the growth of transformants carrying DdaFIG_1Ri, DdaFIG_2Ri, and DdaFIG_1,2Ri was significantly enhanced in the presence of Congo red and SDS (Figure 5A,B), suggesting the involvement of *DdaFIG_1* and *DdaFIG_2* in regulating cell wall stress resistance, presumably by regulating the expression of *β*-1,3-glucose synthase genes and those involved in lipid metabolism.

NaCl, KCl, and sorbitol were used to evaluate their involvement in responding to hyperosmotic stress. Suppression of *DdaFIG_1* did not affect the growth response to KCl (Figure 5D) and sorbitol (Figure 5E), while suppression of *DdaFIG_2* and suppression of *DdaFIG_1* and *DdaFIG_2* significantly enhanced growth (Figure 5D,E), suggesting the importance of *DdaFIG_2* in regulating growth in the presence of KCl and sorbitol. However, suppression of *DdaFIG_1* and *DdaFIG_2* individually significantly decreased their ability to handle NaCl stress, whereas suppression of both genes greatly improved the tolerance to NaCl (Figure 5C).

H_2_O_2_ was used to assess the role of these genes in responding to oxidative stress. The sensitivity to different concentrations of H_2_O_2_ was not affected by knocking down *DdaFIG_1*, while knocking down *DdaFIG_2* singly or simultaneously with *DdaFIG_1* increased the resistance to H_2_O_2_ (Figure 5F). Growth at a low-temperature (15 °C) suggested that both genes participate in managing low-temperature stress (Figure 5, which is consistent with the observation in *A. oligospora* [20].

## 4. Discussion

The FIG_1 protein is involved in various cellular processes in filamentous fungi. This paper identified two genes homologous to FIG_1 in the mechanical-trap forming *D. dactyloides* (Figure 1) and characterized their function in the trap formation process (Figure 4) using RNAi (Figure 2 and Figure S1). A similar result was also found in 3-D adhesive traps forming *A. oligospora* [20] and 2-D-trap forming *D. haptotyla* [21], indicating the involvement and common mechanism of LACS in the trap formation process in NTF. Both DdaFIG_1 and DdaFIG_2 participate in vegetative growth, conidiation, stress response, and CR formation and inflation (Figure 3, Figure 4 and Figure 5). Aside from their shared functions, DdaFIG_1 and DdaFIG_2 seem to perform some gene-specific functions (Figure 5D–F). Their structural and pI differences may contribute to distinction functions.

Knockdown of *DdaFIG_1* or *DdaFIG_2* impaired mycelial growth (Figure 3). Similar phenotypes have been observed in other NTF *A. oligospora* [20] and *D. haptotyla* [21] and non-NTF *F. graminearum* [8], *C. albicans* [19], and *A. nidulans* [10]. Conidia production ability was decreased in *D. dactyloides* by gene suppression of *DdaFIG_1* or *DdaFIG_2.* Conidiation was also affected in NTF *A. oligospora* [20] and *D. haptotyla* [21] and non-NTF *A. nidulans* [10] when the gene was disrupted or suppressed. These results suggest that the function of *FIG_1* is conserved. Responses to cell wall stress and low-temperature stress were also found to involve FIG_1 in *D. dactyloides*, *A. oligospora* [20], and *D. haptotyla* [21]. Responses of *FIG_2* knockdown mutants to abiotic stressors of cell wall integrity, osmotic, and oxidative stresses are similar between *D. dactyloides* and *D. haptotyla* [21], while the responses to low-temperature stress are different, which may be a result of the off-target effect of RNA interference technology. The *FIG_1/2* single or double knockdown strains have faster a growth rate than the WT strains on PDA plates under some stresses, indicating that LACS responses to calcium concentration may affect other ions absorption and slightly inhibit the mycelium of WT strains. Knocking down *FIG_1/2* genes may alleviate the suppression of other ions absorption by LACS or activate other responses through adjusting the intracellular concentration of calcium. The underlying mechanism remains to be investigated.

Many genes have been identified to be involved in the predatory process of 3-D adhesive traps forming *A. oligospora*, such as the autophagy-related gene *atg8* [29], peroxisome biogenesis genes *AoPEX1* and *AoPEX6* [30], mitogen-activated protein kinases (MAPK) *Hog1* and *Msb2* [31], G-protein β-subunit *Gpb1* [32], and the others [33,34,35,36,37,38]. However, the genes involved in the predatory process of NTF forming 2-D adhesive traps or mechanical traps are not well investigated. The *FIG_1* gene is present in NTF and non-NTF, while *FIG_2* is present only in NTF, suggesting that *FIG_2* may be specialized for the predation process of NTF. Such a role of the LACS has been investigated in the 3-D-trap forming *A. oligospora* [20], the 2-D-trap forming *D. haptotyla* [21], and the mechanical-trap forming *D. dactyloides* in this study, indicating that the LACS is a vital component of the predatory lifestyle. To comprehensively understand how the LACS and its components regulate the predatory process, and how *FIG_1* and *FIG_2* exert shared and gene-specific functions, other genes and processes involved in the calcium signaling pathway should be investigated in diverse NTF. This investigation will require a multiple-pronged approach, including but not limited to gene deletion, monitoring the subcellular localization of Ca^2+^, and transcriptomic analysis. In addition, since the LACS likely interacts with other Ca^2+^ channels, transporters, pumps, and Ca^2+^-binding proteins, their spatial and temporal dynamics under diverse conditions should also be investigated.

The intracellular Ca^2+^ levels in yeast cells are regulated by calcium transporters and channels in the cell membrane and organelle membranes, including two of the HACS located in the cell membrane *Cch1* and *Mid1* [39], three located in the vacuolar membrane, *Ycx1*, *Vcx1*, and *Pmc1* [40], three located in the endoplasmic reticulum (ER) membrane, *Eca1*, *Spf1*, and *Cmr1* [41], and one located in the Golgi membrane, *Pmr1* [42]. To prevent the deleterious effects of excessive free Ca^2+^ in the cytoplasm, the excess of Ca^2+^ is rapidly eliminated using Ca^2+^ pumps and exchangers. Cytosolic Ca^2+^ in fungi cells is maintained at the 50~200 nM level [43]. Calcium can be stored in vacuoles (2 mM), mitochondria (140~400 nM), and the secretory compartments of ER (10 mM) or Golgi (300 mM) [44]. In addition, some Ca^2+^ are pumped out of the cell. Some of the intracellular Ca^2+^ can be released from the internal stores to the cytoplasm [45]. As noted in the Introduction, dynamic cytoplasmic Ca^2+^ changes regulate the location and activity of diverse proteins, including the calcium binding proteins calmodulin (CaM) and the serine/threonine phosphatase calciuneurin [46]. Resulting signals through the CaM/calcineurin pathway regulate a transcription factor, the calcineurin-responsive zinc finger protein CRZ [47]. Dephosphorylation of CRZ causes its immediate entry in the nucleus to activate the transcription of a range of genes in diverse pathways [47].

## 5. Conclusions

Two genes homologous to *FIG_1,* the only component of LACS in most fungi, were identified in *D. dactyloides*, a CR-forming nematode trapping fungus, and their functions were characterized using RNAi. Both *DdaFIG_1* and *Dda-FIG_2* play important roles in vegetative growth, conidiation, stress response, CR formation, and the predation of nematodes, confirming that the LACS plays multiple critical roles in diverse NTF.

## Data Availability

Not applicable.

## Supplementary Materials

The following supporting information can be downloaded at: https://www.mdpi.com/article/10.3390/jof9100975/s1, Figure S1: Schematic diagram of *DdaFIG_1* and *DdaFIG_2* RNA interference. Figure S2: Colony morphology of WT and transformants containing DhFIG_1Ri, DhFIG_2Ri, and DhFIG_1, 2Ri under diverse abiotic stresses. Table S1: Primers used in this study. Table S2: Relative expression levels of *DdaFIG_1* in transformants carrying the DdaFIG_1 RNAi construct. Table S3: Relative expression levels of *DdaFIG_2* in transformants carrying the *DdaFIG_2* RNAi construct. Table S4: Relative expression levels of *DdaFIG_1* and *DdaFIG_2* in transformants carrying the DdaFIG_1,2 RNAi construct. Table S5: Conidiation (conidia numbers) of the wild-type strain and transformants containing individual RNAi constructs. Table S6: Numbers of the trap formed by the wild-type strain and transformants carrying individual RNAi constructs. Table S7: Inflated traps (%) formed by the wild-type strain and transformants carrying individual RNAi constructs.
