# Supplementary material for: Multiple Roles of the Low-Affinity Calcium Uptake System in Drechslerella dactyloides, a Nematode-Trapping Fungus That Forms Constricting Rings
Source: J Fungi (Basel). 2023 Sep 28;9(10):975. doi: 10.3390/jof9100975 (PMC10607529; doi:10.3390/jof9100975)
Supplement: Supplementary file 1 [file jof-09-00975-s001.zip › jof-2606000-supplementary.pdf]

Supplementary Materials

Article

Multiple Roles of the Low-Affinity Calcium Uptake System in *Drechslerella dactyloides*, a Nematode-Trapping Fungus That Forms Constricting Rings

Xiaozhou Zhao [1], Yani Fan [2,3], Liao Zhang [1], Weiwei Zhang [2,3], Meichun Xiang [2,3], Seogchan Kang [4], Shunxian Wang [1,*] and Xingzhong Liu [1,*]

[1] State Key Laboratory of Medicinal Chemical Biology, Key Laboratory of Molecular Microbiology and Technology of the Ministry of Education, Department of Microbiology, College of Life Science, Nankai University, Tianjin 300071, China
[2] State Key Laboratory of Mycology, Institute of Microbiology, Chinese Academy of Sciences, Beijing 100101, China
[3] University of Chinese Academy of Sciences, Beijing 100049, China
[4] Department of Plant Pathology & Environmental Microbiology, The Pennsylvania State University, University Park, PA 16802, USA; sxk55@psu.edu
* Correspondence: shunxianwang@nankai.edu.cn (S.W.); liuxz@nankai.edu.cn (X.L.)

Table S1 Primers used in this study

Primers	Sequence (5′ to 3′)
HYG-540F	TTGCAAGACCTGCCTGAAACCGAACTGCCC
HYG-540R	AACCAAGCTCTGATAGAGTTGGTCAAGACC
Tublin-RT-F	CAAGGTCAGCATGAAGGAG
Tublin-RT-R	CGATAATGAGGAGGAAGGT
DdaFIG_1-RT-F	GATGCCGAGTCGATTCGTTTC
DdaFIG_1-RT-R	CACCAACAAGCCAATCGTTAC
DdaFIG_2-RT-F	TCCGCATCGACGACCACTATG
DdaFIG_2-RT-R	GATCCGCACAGCCATTGCGA

Table S2 Relative expression levels of *DdaFIG_1* in transformants carrying the *DdaFIG_1* RNAi construct

Target	Sample	Control	Expression	Expression SEM	Corrected Expression SEM	Mean Cq	Cq SEM
DdaFIG_1	WT	*	1.00000	0.07212	0.07212	24.76	0.09217
DdaFIG_1	*DdaFIG_2Ri-25*		0.49172	0.09128	0.09128	29.14	0.02594
DdaFIG_1	*DdaFIG_2Ri-29*		0.53781	0.03280	0.03280	27.85	0.08963
DdaTublin	WT	*				18.11	0.58704
DdaTublin	*DdaFIG_2Ri-25*					21.45	0.10040
DdaTublin	*DdaFIG_2Ri-29*					20.30	0.05672

Table S3 Relative expression levels of *DdaFIG_2* in transformants carrying the *DdaFIG_2* RNAi construct

Target	Sample	Control	Expression	Expression SEM	Corrected Expression SEM	Mean Cq	Cq SEM
DdaFIG_2	WT	*	1.00000	0.08365	0.08365	21.79	0.08365
DdaFIG_2	*DdaFIG_2Ri-31*		0.51723	0.05525	0.05525	27.17	0.16000
DdaFIG_2	*DdaFIG_2Ri-46*		0.58854	0.03640	0.03640	25.55	0.09073
DdaTublin	WT	*				16.78	0.45740
DdaTublin	*DdaFIG_2Ri-31*					21.20	0.03771
DdaTublin	*DdaFIG_2Ri-46*					19.77	0.06381

Table S4 Relative expression levels of *DdaFIG_1* and *DdaFIG_2* in transformants carrying the *DdaFIG_1,2* RNAi construct

Target	Sample	Control	Expression	Expression SEM	Corrected Expression SEM	Mean Cq	Cq SEM
DdaFIG_1	WT	*	1.00000	0.07212	0.07212	24.76	0.09217
DdaFIG_1	*DdaFIG_1,2Ri-16*		0.51388	0.03892	0.03892	25.52	0.11150
DdaFIG_1	*DdaFIG_1,2Ri-27*		0.48963	0.05697	0.05697	26.63	0.16623
DdaTublin	WT	*				18.11	0.58704
DdaTublin	*DdaFIG_1,2Ri-16*					17.91	0.41693
DdaTublin	*DdaFIG_1,2Ri-27*					18.94	0.23892
DdaFIG_2	WT	*	1.00000	0.08365	0.08365	21.79	0.08365
DdaFIG_2	*DdaFIG_1,2Ri-16*		0.53811	0.03960	0.03960	24.50	0.10786
DdaFIG_2	*DdaFIG_1,2Ri-27*		0.46459	0.05281	0.05281	24.02	0.16623
DdaTublin	WT	*				16.78	0.45740
DdaTublin	*DdaFIG_1,2Ri-16*					18.59	0.34042
DdaTublin	*DdaFIG_1,2Ri-27*					17.00	0.28377

Table S5 Conidiation (conidia numbers) of the wild type strain and transformants containing individual RNAi constructs

Strain	Rep1	Rep2	Rep3	Rep4	Rep5	Rep6	Rep7	Average	S.D.
WT	90000	83000	76000	95000	81000	72000	77000	82000	8124
DdaFIG_1Ri-25	36000	38000	42000	42000	40000	33000	45000	39429	4077
DdaFIG_1Ri-29	35000	42000	39000	40000	34000	45000	35000	38571	4117
DdaFIG_2Ri-31	42000	33000	37000	32000	40000	44000	35000	37571	4577
DdaFIG_2Ri-46	38000	36000	42000	41000	40000	43000	32000	38857	3848
DdaFIG_1,2Ri-16	16000	12000	15000	15000	17000	11000	14000	14286	2138
DdaFIG_1,2Ri-27	15000	18000	14000	13000	10000	12000	15000	13857	2545

Table S6 Numbers of the trap formed by the wild type strain and transformants carrying individual RNAi constructs

Strain	Rep1	Rep2	Rep3	Rep4	Rep5	Rep6	Rep7	Average	S.D.
16 h after induction by nematodes									
WT	124	150	148	135	132	118	124	133	12
DdaFIG_1Ri-25	88	90	89	78	66	96	75	83	10
DdaFIG_1Ri-29	59	60	72	95	85	86	73	75	4
DdaFIG_2Ri-31	55	70	57	58	65	76	72	65	8
DdaFIG_2Ri-46	76	70	69	79	60	73	73	71	6
DdaFIG_1,2Ri-16	39	52	55	48	53	46	50	49	5
DdaFIG_1,2Ri-27	49	56	63	57	47	58	60	56	6
24 h after induction by nematodes									
WT	200	187	176	215	208	170	192	193	16
DdaFIG_1Ri-25	110	101	127	109	99	97	119	109	11
DdaFIG_1Ri-29	132	122	106	100	120	133	105	117	13
DdaFIG_2Ri-31	128	128	97	107	115	130	123	118	13
DdaFIG_2Ri-46	110	98	130	92	108	115	112	110	12
DdaFIG_1,2Ri-16	79	87	75	80	76	69	74	77	6
DdaFIG_1,2Ri-27	65	63	80	85	78	70	68	73	8

Table S7 Inflated traps (%) formed by the wild type strain and transformants carrying individual RNAi constructs

Strain	Rep1	Rep2	Rep3	Rep4	Rep5	Rep6	Rep7	Average	S.D.
16 h after induction by nematodes									
WT	5.65	6.67	7.09	5.56	6.25	7.63	5.44	6.33	0.84
DdaFIG_1Ri-25	3.12	3.61	4.21	5.13	4.92	4.17	5.33	4.36	0.82
DdaFIG_1Ri-29	3.39	4.58	6.25	2.37	3.82	5.23	5.14	4.40	1.30
DdaFIG_2Ri-31	4.55	5.71	3.95	2.16	3.08	2.30	2.78	3.50	1.30
DdaFIG_2Ri-46	3.29	2.86	3.62	4.11	3.75	3.08	2.40	3.30	0.58
DdaFIG_1,2Ri-16	5.13	2.40	1.36	2.60	2.83	2.17	2.50	2.71	1.16
DdaFIG_1,2Ri-27	3.06	2.68	2.78	1.75	3.72	2.16	2.92	2.72	0.63
24 h after induction by nematodes									
WT	15.38	14.71	14.63	16.98	16.47	15.74	16.28	15.74	0.89
DdaFIG_1Ri-25	12.73	12.13	12.20	11.01	11.62	13.40	11.97	12.15	0.77
DdaFIG_1Ri-29	11.17	11.68	9.20	13.00	10.21	12.41	12.62	11.47	1.38
DdaFIG_2Ri-31	9.96	9.77	10.31	7.94	8.70	7.69	9.35	9.10	1.02
DdaFIG_2Ri-46	10.00	11.48	12.31	8.70	9.95	8.26	9.15	9.98	1.47
DdaFIG_1,2Ri-16	8.23	4.89	6.67	9.38	7.57	7.97	8.11	7.54	1.42
DdaFIG_1,2Ri-27	8.46	7.94	7.81	8.82	8.33	7.50	5.88	7.82	0.96

Table S8 Growth rate (colony diameter (mm)) of wild type (WT) strain and the mutants under cell wall stresses

Strain	Rep1	Rep2	Rep3	Rep4	Rep5	Rep6	Rep7	Average	S.D.
**14-day-cultivation PDA**									
WT	37.6	38	36.9	37.2	37.5	37.5	38.2	37.56	0.443
DdaFIG_1Ri-25	32.5	33.6	32.8	33.2	33.3	32.9	33	33.04	0.360
DdaFIG_1Ri-29	33.4	32.8	33	33.5	32.8	33.8	33	33.19	0.385
DdaFIG_2Ri-31	32.8	33.5	33.4	33.6	32.8	31.9	33.6	33.09	0.628
DdaFIG_2Ri-46	33.1	33.5	32.9	33	34	33.8	33	33.33	0.439
DdaFIG_1,2Ri-16	25.7	26.5	27.1	26.6	25.9	26.2	26.8	26.40	0.497
DdaFIG_1,2Ri-27	26.5	27	27.5	25.8	26.2	25.8	26.4	26.46	0.621
**PDA containing 0.1 mg/mL Congo red**									
WT	36.8	35.2	37.1	36.5	35.9	36	36.4	36.27	0.632
DdaFIG_1Ri-25	35.8	35.6	36.1	36.5	35.7	35.8	36.2	35.96	0.321
DdaFIG_1Ri-29	36.2	36.3	35.8	36.2	35.7	36.2	35.9	36.04	0.237
DdaFIG_2Ri-31	36.2	36	35.9	36.5	35.7	35.8	36	36.01	0.267
DdaFIG_2Ri-46	36	36.4	35.8	36.3	36.2	35.7	35.8	36.03	0.275
DdaFIG_1,2Ri-16	37	36.9	37.2	37.3	38	37.8	36.5	37.24	0.519
DdaFIG_1,2Ri-27	36.3	36.6	36.5	35.6	37	37.1	36.3	36.49	0.501
**PDA containing 0.2 mg/mL Congo red**									
WT	34.2	33.5	33.9	34.4	35	34.2	34	34.17	0.464
DdaFIG_1Ri-25	34.2	35.1	34.2	35	34.9	35	35.2	34.8	0.42
DdaFIG_1Ri-29	35.5	35.2	35.9	35.6	35.3	35.4	34.8	35.39	0.344
DdaFIG_2Ri-31	34.5	35.1	33.8	34	34.6	35.1	35	34.59	0.527
DdaFIG_2Ri-46	36	36.4	35.8	36.3	36.2	35.7	35.8	36.03	0.275
DdaFIG_1,2Ri-16	37	36.9	37.2	37.3	38	37.8	36.5	37.24	0.519
DdaFIG_1,2Ri-27	35.8	35	34.8	35	35.2	35.8	34.8	35.2	0.432
**PDA containing 0.4 mg/mL Congo red**									
WT	33.1	32.1	32	31.9	32	33	32.4	32.36	0.5
DdaFIG_1Ri-25	33.1	33.5	32.4	33.3	33.9	32.3	32.6	33.01	0.601
DdaFIG_1Ri-29	32.8	32.5	33	31.9	32.5	32.4	32.5	32.51	0.344
DdaFIG_2Ri-31	30.2	30.9	31	31.2	31.4	31.5	32	31.17	0.562
DdaFIG_2Ri-46	31.5	31.9	32	32.2	33.3	32	31	31.99	0.706
DdaFIG_1,2Ri-16	33.6	34.5	33.8	34	33.7	33.4	32.8	33.69	0.523
DdaFIG_1,2Ri-27	32.8	33	32.7	33.4	33.5	32.7	33	33.01	0.324
**PDA containing 0.01% SDS**									
WT	19.6	19.5	21	19	20	19.5	18.9	19.64	0.704
DdaFIG_1Ri-25	20	19.3	19.4	18.5	18.6	19	19.5	19.19	0.527
DdaFIG_1Ri-29	19.5	18.6	19.8	20.3	19.5	18.6	18.3	19.23	0.739
DdaFIG_2Ri-31	17.5	17	18.3	18.5	19.1	19.2	18.6	18.31	0.807
DdaFIG_2Ri-46	18.7	18.5	18.8	17.8	19.2	19	18.4	18.63	0.457
DdaFIG_1,2Ri-16	20.9	22	21.8	20.5	20.4	20.5	20.3	20.91	0.701
DdaFIG_1,2Ri-27	19.5	19.6	20.5	20	20.5	21	19.3	20.06	0.629

PDA containing 0.02% SDS

WT	15.6	15.4	14.7	14.8	15	15.8	15.1	15.2	0.412
DdaFIG_1Ri-25	15	14.9	15.2	15.3	15	14.7	15.2	15.04	0.207
DdaFIG_1Ri-29	15.4	15.4	15.3	14.8	14.9	15.6	15	15.2	0.3
DdaFIG_2Ri-31	14.8	14.8	14.5	15	14.4	14.7	15.2	14.77	0.275
DdaFIG_2Ri-46	14.7	15	13.9	14.8	14.8	14.5	14.6	14.61	0.353
DdaFIG_1,2Ri-16	17.6	18.2	17.6	18.5	18	18.6	18.4	18.13	0.411
DdaFIG_1,2Ri-27	17.8	17.5	16.8	16.6	18.2	16.5	17.2	17.23	0.640

Table S9 Growth rate (colony diameter (mm)) of wild type (WT) strain and the mutants under osmotic stresses

Strain	Rep1	Rep2	Rep3	Rep4	Rep5	Rep6	Rep7	Average	S.D.
**14-day-cultivation PDA**									
WT	34.5	36.3	35.4	35	34.7	35.5	35.9	35.33	0.645
DdaFIG_1Ri-25	32.3	31.6	31.9	31.4	32.1	31.5	30.5	31.61	0.59
DdaFIG_1Ri-29	32.1	31	32	30.9	31.2	30.4	31.5	31.3	0.611
DdaFIG_2Ri-31	31.4	33	32.4	31.8	31.5	32	31.5	31.94	0.583
DdaFIG_2Ri-46	32	31.8	32.2	31.9	31.7	32.5	32.4	32.07	0.304
DdaFIG_1,2Ri-16	26.4	26.8	25.7	26	25.5	25.6	25.7	25.96	0.479
DdaFIG_1,2Ri-27	25.6	26.5	26.8	26.1	25.5	26.2	25.2	25.99	0.576
**PDA containing 0.1 M NaCl**									
WT	29	28.9	28	27.8	28.4	29.1	28.5	28.53	0.502
DdaFIG_1Ri-25	22.4	22.5	23	22.8	23.6	23.6	23.5	23.06	0.516
DdaFIG_1Ri-29	22.4	23	24.1	23.8	23.9	22.2	22.5	23.13	0.795
DdaFIG_2Ri-31	24.5	24.5	23.9	23.2	23	22.8	23.7	23.66	0.69
DdaFIG_2Ri-46	23.9	24	24.2	23.5	22.9	23.1	22.7	23.47	0.585
DdaFIG_1,2Ri-16	25.3	24.3	24.3	24.7	23.8	24.5	23.8	24.39	0.524
DdaFIG_1,2Ri-27	24.5	24.6	24.7	25.1	25	23.5	24	24.49	0.564
**PDA containing 0.2 M NaCl**									
WT	19.4	18.9	19.1	19.1	19.1	18.8	19.3	19.1	0.208
DdaFIG_1Ri-25	14.2	14.3	14	15.1	15.2	14.3	15	14.59	0.495
DdaFIG_1Ri-29	15.2	14.7	14.6	16.2	14.2	14.4	14	14.76	0.744
DdaFIG_2Ri-31	14.6	14.9	15	14.5	15.2	14.5	16	14.96	0.532
DdaFIG_2Ri-46	16.2	15.4	14.5	14.7	15	14.2	14	14.86	0.757
DdaFIG_1,2Ri-16	16.8	15.7	17.6	18.3	17.5	16.9	17.4	17.17	0.816
DdaFIG_1,2Ri-27	17.2	17.5	16.5	16.8	18	16.2	16	16.89	0.722
**PDA containing 0.3 M NaCl**									
WT	13.9	13.6	13.8	13.6	13.7	13.5	14	13.72	0.18
DdaFIG_1Ri-25	12.5	12.9	13.2	13.2	12.6	12.7	12.5	12.8	0.306
DdaFIG_1Ri-29	13	12.8	13.1	13.2	13.5	12.4	12	12.86	0.509
DdaFIG_2Ri-31	10.5	11.5	11	10.9	10.6	10.8	11.2	10.93	0.345
DdaFIG_2Ri-46	11.6	11.5	10.5	10.6	10.8	10.7	11	10.96	0.435
DdaFIG_1,2Ri-16	13.7	12.5	12.5	13	12.6	13.1	12.7	12.87	0.435
DdaFIG_1,2Ri-27	13.3	12.9	13	12.8	12.5	12.7	13.1	12.9	0.265
**PDA containing 0.1 M KCl**									
WT	29.6	28.7	29.1	30.3	28.7	29.2	28.7	29.19	0.596
DdaFIG_1Ri-25	25.8	26.9	26.9	27	26.5	26.7	26.5	26.61	0.38
DdaFIG_1Ri-29	25.8	25.7	26.7	26.5	26.2	26	26.8	26.24	0.403
DdaFIG_2Ri-31	27.8	28.5	28.2	29	28.6	29.1	28.3	28.5	0.421
DdaFIG_2Ri-46	29	28.5	28.9	27.4	27.6	29.3	28.6	28.47	0.663
DdaFIG_1,2Ri-16	24.5	23.6	22.8	23.5	23.3	23.4	23.2	23.47	0.452
DdaFIG_1,2Ri-27	22.9	23.2	24	22.6	23.8	23	22.6	23.16	0.48
**PDA containing 0.2 M KCl**									

WT	21	21.7	21.2	20.9	20.9	21.2	20.5	21.06	0.369
DdaFIG_1Ri-25	18.5	19	20.2	18.7	17.6	18.1	18.4	18.64	0.756
DdaFIG_1Ri-29	17.6	18	18.8	19	18.9	18.3	17.8	18.34	0.523
DdaFIG_2Ri-31	23	23.5	22.9	22.8	23.1	22.5	22.7	22.93	0.296
DdaFIG_2Ri-46	23	23.1	23.5	22.8	22.7	23.2	22.3	22.94	0.368
DdaFIG_1,2Ri-16	20	20.4	20.4	20.7	19.8	20	19.4	20.1	0.377
DdaFIG_1,2Ri-27	20.2	19.5	19.6	20.4	19.5	20	20.3	19.93	0.338
**PDA containing 0.3 M KCl**									
WT	14.6	14.2	15	15.1	14.8	14.5	15.5	14.81	0.43
DdaFIG_1Ri-25	12.6	13.4	14	12.5	12	13	12.9	12.91	0.601
DdaFIG_1Ri-29	13.4	13.3	12.9	12.5	13	12.5	13.2	12.97	0.337
DdaFIG_2Ri-31	15.6	15.5	15.8	15.9	15.5	16.2	15.8	15.76	0.232
DdaFIG_2Ri-46	14.9	15.5	16.2	16	15.4	14.8	15.9	15.53	0.501
DdaFIG_1,2Ri-16	14.3	13.8	15.2	14.1	14.5	13.8	14.2	14.27	0.417
DdaFIG_1,2Ri-27	14.3	13.5	14.2	14.2	13.9	14.5	13.7	14.04	0.308
**PDA containing 0.2 M Sorbitol**									
WT	31.4	32.5	30.9	31.4	30.5	31.5	31.6	31.4	0.622
DdaFIG_1Ri-25	28.2	27.8	28.7	28.8	27.5	28	28.3	28.19	0.432
DdaFIG_1Ri-29	27.9	28.5	28.7	29	28.3	27.5	28.3	28.31	0.461
DdaFIG_2Ri-31	30.5	29.7	28.8	30.7	30	29.6	29.2	29.79	0.623
DdaFIG_2Ri-46	30.1	29.2	28.5	29.5	31	29.3	30.2	29.69	0.755
DdaFIG_1,2Ri-16	26.4	25.8	26.4	26.5	26.7	25.9	26.5	26.31	0.309
DdaFIG_1,2Ri-27	25.5	26.2	26.5	26	26.5	26.2	26.8	26.24	0.389
**PDA containing 0.5 M Sorbitol**									
WT	28.5	27.2	28.1	28.2	28.5	27.9	27.1	27.93	0.574
DdaFIG_1Ri-25	26.3	25.8	25.3	26	25	25.3	24.9	25.51	0.489
DdaFIG_1Ri-29	26.2	25.7	24.5	25.5	26.6	24.6	24.2	25.33	0.848
DdaFIG_2Ri-31	27.6	27.6	26.9	27.5	28.1	27.9	27.5	27.56	0.348
DdaFIG_2Ri-46	26.9	27.3	27	28	26.6	27.8	27.9	27.36	0.51
DdaFIG_1,2Ri-16	23.9	25.1	23.4	24.1	25.1	23.8	24.4	24.26	0.602
DdaFIG_1,2Ri-27	24	24.3	25.2	24	23.8	23.6	23.8	24.1	0.493
**PDA containing 0.8 M Sorbitol**									
WT	24.3	24.2	25.4	25.6	24.3	24.5	25.7	24.86	0.675
DdaFIG_1Ri-25	21.8	22.8	23.3	21.2	21.4	22.2	21.5	22.03	0.723
DdaFIG_1Ri-29	21.6	21.2	22.3	21.8	21	22	21.1	21.57	0.456
DdaFIG_2Ri-31	24.5	25.1	25.1	24.7	25.2	23.8	24.5	24.7	0.457
DdaFIG_2Ri-46	23.8	24.5	25	25	24.2	24.9	25.4	24.69	0.508
DdaFIG_1,2Ri-16	24.1	23.5	24.3	22.8	23.5	24.2	23.9	23.76	0.489
DdaFIG_1,2Ri-27	23.5	23.7	22.8	23.4	23.3	22.8	24.1	23.37	0.433

Table S10 Growth rate (colony diameter (mm)) of wild type (WT) strain and the mutants under oxidative stress

Strain	Rep1	Rep2	Rep3	Rep4	Rep5	Rep6	Rep7	Average	S.D.
**14-day-cultivation PDA**									
WT	37.6	38	36.9	37.2	37.5	37.5	38.2	37.56	0.443
DdaFIG_1Ri-25	32.5	33.6	32.8	33.2	33.3	32.9	33	33.04	0.360
DdaFIG_1Ri-29	33.4	32.8	33	33.5	32.8	33.8	33	33.19	0.385
DdaFIG_2Ri-31	32.8	33.5	33.4	33.6	32.8	31.9	33.6	33.09	0.628
DdaFIG_2Ri-46	33.1	33.5	32.9	33	34	33.8	33	33.33	0.439
DdaFIG_1,2Ri-16	25.7	26.5	27.1	26.6	25.9	26.2	26.8	26.40	0.497
DdaFIG_1,2Ri-27	26.5	27	27.5	25.8	26.2	25.8	26.4	26.46	0.621
**PDA containing 0.001% H_2O_2**									
WT	32.3	32.2	32.8	31.9	32	31.5	33.8	32.36	0.695
DdaFIG_1Ri-25	28	27.8	28	26.2	28.5	27.5	28.6	27.8	0.743
DdaFIG_1Ri-29	27.5	28.5	28	29.1	28.3	29.3	26.9	28.23	0.787
DdaFIG_2Ri-31	34.6	33.5	32.3	32	32.8	33.5	32	32.96	0.893
DdaFIG_2Ri-46	32.8	33.4	32.8	34	33.7	33.6	34.2	33.5	0.504
DdaFIG_1,2Ri-16	31	30.8	31.5	30	31.4	30.6	31.8	31.01	0.567
DdaFIG_1,2Ri-27	30.6	30.5	31.6	30.5	30	30.4	29.6	30.46	0.57
**PDA containing 0.003% H_2O_2**									
WT	30	31	30.8	29.2	30.4	30.4	29.6	30.2	0.595
DdaFIG_1Ri-25	26.4	25.7	27.5	27	25.8	26.3	25	26.24	0.776
DdaFIG_1Ri-29	26.6	26.7	27.4	26	27.2	26.5	25.6	26.57	0.582
DdaFIG_2Ri-31	28.1	28	27.5	28	29.4	29.6	28.5	28.44	0.723
DdaFIG_2Ri-46	28.9	27.5	29	29.4	31	28.5	29.2	29.07	0.976
DdaFIG_1,2Ri-16	27.4	28.6	28.2	27.9	28.4	28.4	28.3	28.17	0.373
DdaFIG_1,2Ri-27	28.2	28.5	28.4	29.2	28	27.8	28	28.3	0.431

Table S11 Growth rate (colony diameter (mm)) of wild type (WT) strain and the mutants under low temperature

Strain	Rep1	Rep2	Rep3	Rep4	Rep5	Rep6	Rep7	Average	S.D.
**14-day-cultivation PDA**									
WT	37.6	38	36.9	37.2	37.5	37.5	38.2	37.56	0.443
DdaFIG_1Ri-25	32.5	33.6	32.8	33.2	33.3	32.9	33	33.04	0.360
DdaFIG_1Ri-29	33.4	32.8	33	33.5	32.8	33.8	33	33.19	0.385
DdaFIG_2Ri-31	32.8	33.5	33.4	33.6	32.8	31.9	33.6	33.09	0.628
DdaFIG_2Ri-46	33.1	33.5	32.9	33	34	33.8	33	33.33	0.439
DdaFIG_1,2Ri-16	25.7	26.5	27.1	26.6	25.9	26.2	26.8	26.40	0.497
DdaFIG_1,2Ri-27	26.5	27	27.5	25.8	26.2	25.8	26.4	26.46	0.621
**Cultivation under 15 °C**									
WT	17.6	18	17.8	17	16.9	17.5	17.2	17.43	0.411
DdaFIG_1Ri-25	19.2	18.5	18.2	20.6	20	20	19.6	19.44	0.864
DdaFIG_1Ri-29	18.2	18.5	19.5	20.2	20.5	19.4	20.4	19.53	0.912
DdaFIG_2Ri-31	16.4	18	16.5	16.3	17	16.3	16.4	16.7	0.622
DdaFIG_2Ri-46	16.7	16.5	16.9	16.4	15.8	17.2	16.5	16.57	0.439
DdaFIG_1,2Ri-16	13.4	13.3	12.9	13.2	13.4	12.5	12.8	13.07	0.345
DdaFIG_1,2Ri-27	13.5	12.6	13.5	12.2	12.6	12.5	13.2	12.87	0.522

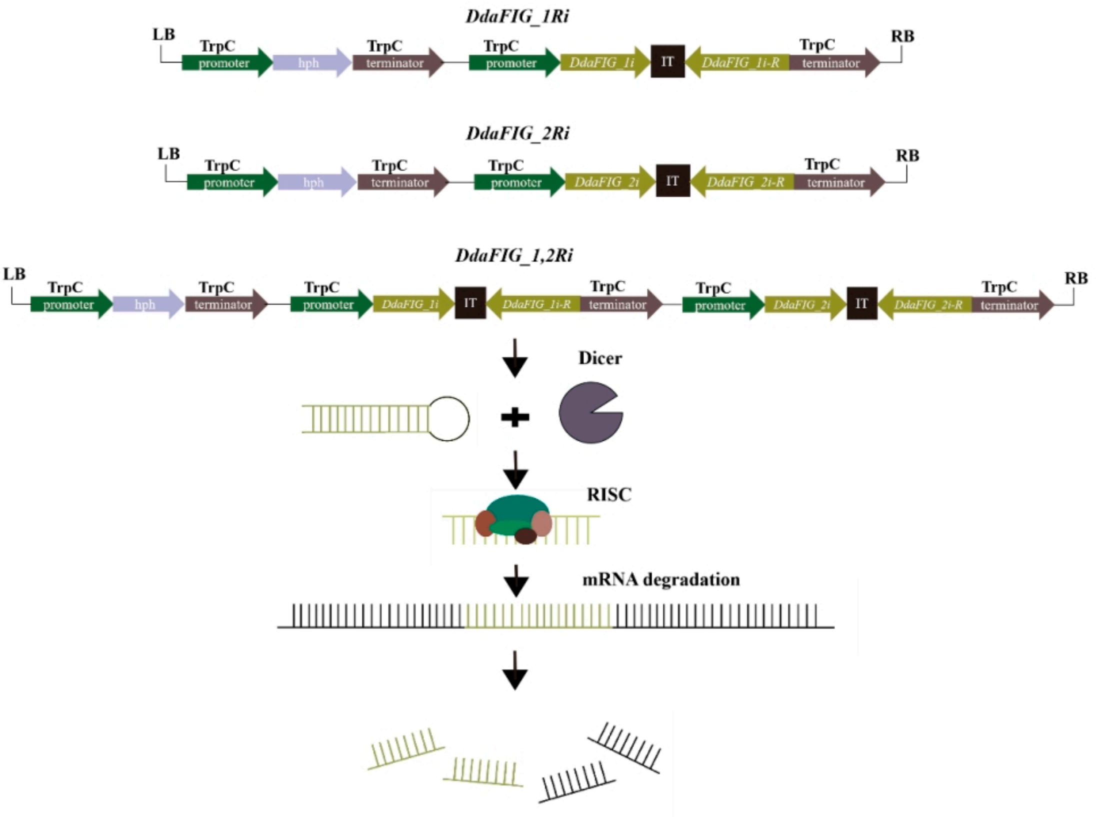

Figure S1. Schematic diagram of the constructs used for suppressing the expression of *DdaFIG_1* and *DdaFIG_2* via RNAi

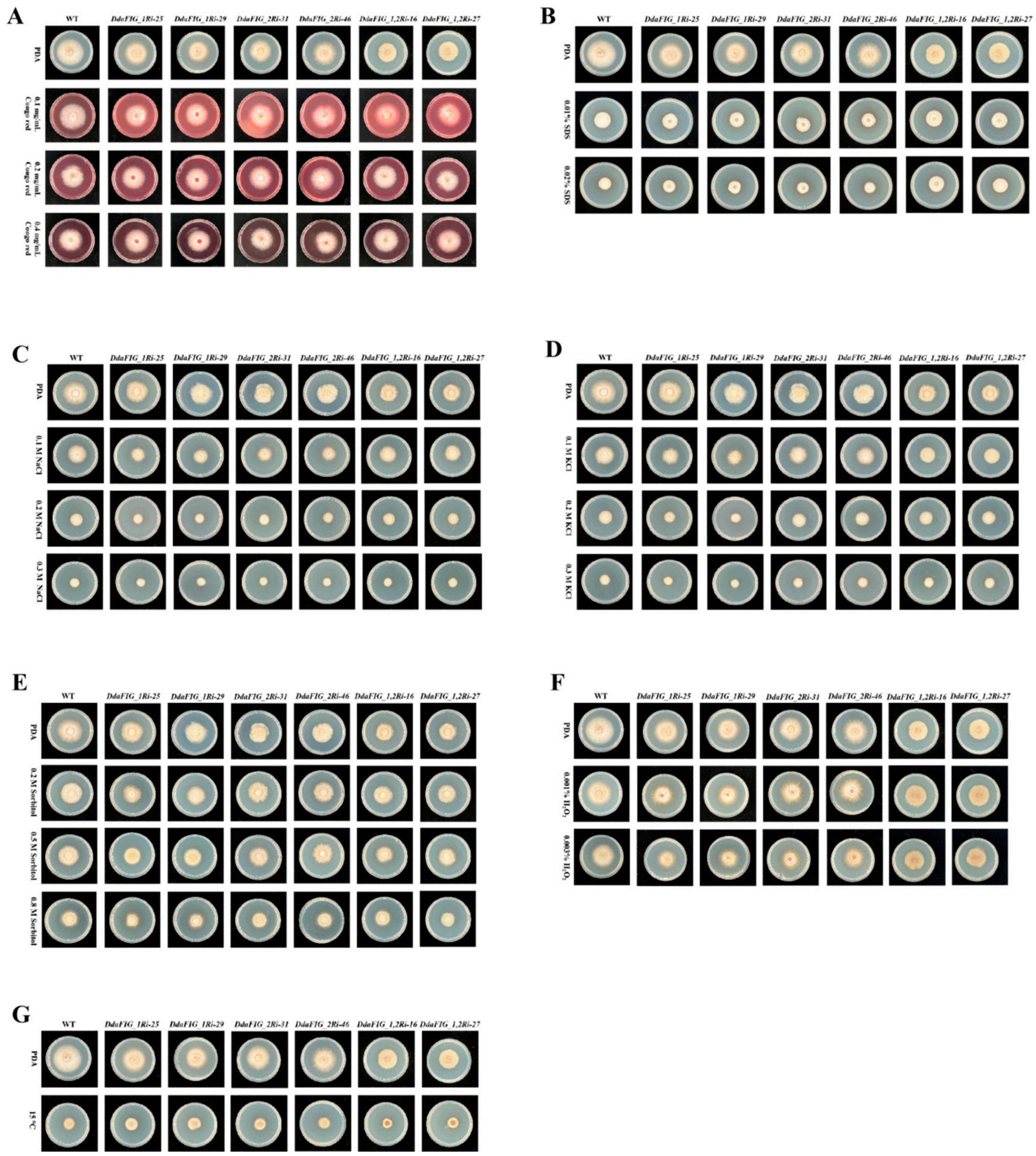

Figure S2. Colony morphology of the WT strain and transformants carrying individual RNAi constructs under diverse abiotic stresses. (A–G) Colony growth and morphology on PDA containing Congo red, SDS, NaCl, KCl, sorbitol, H_2O_2 and growth at 15 °C. ** means *p*-values < 0.01, two-tailed *t*-test, n = 7.
